# Inflammation-Based Scores Increase the Prognostic Value of Circulating Tumor Cells in Primary Breast Cancer

**DOI:** 10.3390/cancers12051134

**Published:** 2020-05-01

**Authors:** Svetlana Miklikova, Gabriel Minarik, Tatiana Sedlackova, Jana Plava, Marina Cihova, Silvia Jurisova, Katarina Kalavska, Marian Karaba, Juraj Benca, Bozena Smolkova, Michal Mego

**Affiliations:** 1 Department of Molecular Oncology, Cancer Research Institute, Biomedical Research Center of the Slovak Academy of Sciences, Dubravska Cesta 9, 845 05 Bratislava, Slovakia; svetlana.miklikova@savba.sk (S.M.); jana.plava@savba.sk (J.P.); marina.cihova@savba.sk (M.C.); bozena.smolkova@savba.sk (B.S.); 2 Department of Molecular Biology, Faculty of Natural Sciences, Comenius University in Bratislava, 841 04 Bratislava, Slovakia; gabriel.minarik@gmail.com (G.M.); tatiana.sedlackova@gmail.com (T.S.); 3 Institute of Molecular Biomedicine, Faculty of Medicine, Comenius University, 841 04 Bratislava, Slovakia; 4 2nd Department of Oncology, Faculty of Medicine, Comenius University and National Cancer Institute, Klenova 1, 833 10 Bratislava, Slovakia; silvia.jurisova@nou.sk (S.J.); katarina.kalavska@nou.sk (K.K.); marian.karaba@nou.sk (M.K.); juraj.benca@nou.sk (J.B.); 5 Department of Medicine, St. Elizabeth University, 811 02 Bratislava, Slovakia

**Keywords:** circulating tumor cells, breast cancer, systemic immune-inflammation index, platelet-to-lymphocyte ratio, neutrophil-to-lymphocyte ratio, monocyte-to-lymphocyte ratio

## Abstract

A correlation between circulating tumor cells (CTCs) and monocytes in metastatic breast cancer (BC), where CTCs and monocyte-to-lymphocyte ratio (MLR) were predictors of overall survival (OS), was recently shown. Herein, we aimed to assess the association between CTCs and the complete blood count (CBC)-derived inflammation-based scores in 284 primary BC patients. CTCs were determined in CD45-depleted peripheral blood mononuclear cells by real time-PCR. This method allowed us to detect a subset of CTCs with an epithelial-to-mesenchymal transition phenotype (CTC EMT), previously associated with inferior outcomes in primary BC. In the present study, CTC EMT positivity (hazard ratio (HR) = 2.4; 95% CI 1.20–4.66, *p* = 0.013) and elevated neutrophil-to-lymphocyte ratio (NLR) (HR = 2.20; 95% CI 1.07–4.55; *p* = 0.033) were associated with shorter progression-free survival (PFS) in primary BC patients. Multivariate analysis showed that CTC EMT-positive patients with NLR ≥ 3 had 8.6 times increased risk of disease recurrence (95% CI 2.35–31.48, *p* = 0.001) compared with CTC EMT-negative patients with NLR < 3. Similarly, disease recurrence was 13.14 times more likely in CTC EMT-positive patients with MLR ≥ 0.34 (95% CI 4.35–39.67, *p* < 0.001). Given its low methodological and financial demands, the CBC-derived inflammation-based score determination could, after broader validation, significantly improve the prognostication of BC patients.

## 1. Introduction

Breast cancer (BC) represents the most frequently diagnosed malignancy and a major cause of death among women. Its histological and molecular characteristics have important implications for therapeutic strategies and prognosis of patients [1]. Traditional prognostic factors include lymph node status, tumor size, histologic type and grade, patient age and ethnicity. Some parameters, originally described as prognostic, e.g., hormonal receptor (HR) status and HER2 overexpression, are currently considered primarily predictive [2]. The markedly diverse clinical outcomes in patients with similar clinicopathological characteristics demonstrate the need for more precise prognostic markers.

Therefore, in recent years research has focused on inflammation, which impacts each step of tumorigenesis [3]. Inflammatory cells engage in a dynamic crosstalk with tumor cells at the primary tumor site, in the circulation, as well as in the lymph nodes or distant metastases [4]. Neutrophils are the most common type of white blood cells and have an important role in the inflammatory response. They become a part of the tumor microenvironment, where their interactions with other cells are crucial for their function [5]. However, their contribution to tumorigenesis is still controversial. Unleashed neutrophilic effectors have been reported to mediate anti-cancer resistance [6,7,8]. Neutrophils, described as N1 neutrophils, can counteract the progression of malignancies through tumor cytotoxicity, tumor rejection, and enhancement of antitumoral immune memory. Recent data showed that neutrophil-driven anti-tumor resistance was dependent on interferon gamma (IFNγ) produced by T cells [9]. Under certain conditions, e.g., after transforming growth factor-β (TGF-β) blockade, neutrophils can gain a pro-inflammatory and pro-tumorigenic phenotype [8]. This suggests that the function they have in cancer may be dictated in a context-dependent fashion [10]. The role of neutrophils as pro-tumorigenic cells, phenotypically denominated as N2 neutrophils, is connected with their impact on angiogenesis and immune surveillance [11,12]. Moreover, it has also been demonstrated that neutrophils can enhance tumor cell dissemination and metastatic seeding in distant organs [13]. However, again, also anti-metastatic neutrophil functions were demonstrated [8,14].

The metastatic process is highly inefficient, as most of the circulating tumor cells (CTCs) die and do not form metastases [3]. Neutrophils were shown to interact with CTCs and expand their metastatic potential by forming CTC–neutrophil clusters via vascular cell adhesion molecule 1 (VCAM-1) [4] and via other mechanisms. Through physical interaction and anchoring of CTCs to the vascular endothelium, they facilitated trans-endothelial migration of tumor cells, extravasation, and formation of new metastases in vivo. Injection of neutrophils in this model significantly increased cancer cell retention [15]. The metastasis process starts with the acquisition of a mesenchymal phenotype via epithelial-to-mesenchymal transition (EMT) followed by intravasation and extravasation processes. CTCs are precursors of metastasis. They travel through the peripheral blood until they reach a desirable metastatic niche at distant organs. Heterogeneous types of CTCs with different phenotypes, biological value, and prognostic potential [16] were detected in the circulation of BC patients [17,18]. The presence of CTCs with an EMT phenotype (CTC EMT) was associated with inferior outcome in primary and metastatic BC [18,19].

Moreover, it has been recently shown that in metastatic BC, CTCs positivity (≥5 CTC in 7.5 mL of peripheral blood) directly correlated with monocyte count, and in combination with a monocyte-to-lymphocyte ratio (MLR) ≥ 0.34, CTCs were significantly associated with decreased overall survival (OS) [20]. Peripheral blood monocytes appear to be recruited into tumors, where they differentiate into tumor-associated macrophages or monocyte-derived dendritic cells. Within the tumor microenvironment, the tumor-educated monocytes can interact with lymphocytes [21]. It has been shown that tumor-induced systemic changes in immune cells, facilitating cancer progression and metastasis, may be mediated by an alteration of the ability of immune cells to respond to cytokines [22]. As monocytes remain in the tissue once they leave the bloodstream, the signaling alterations must develop at the site of their origin (bone marrow or spleen) caused by cancer-induced distant effects. Dysregulated IFNγ signaling in peripheral blood monocytes at diagnosis was identified in BC patients who later relapsed. These results demonstrate that tumor-induced systemic immune changes are evident in the circulation of BC patients [22]. Moreover, other alterations in complete blood count (CBC)-derived inflammation-based scores, e.g., platelet-to-lymphocyte ratio (PLR), systemic immune-inflammation index (SII), and others, were linked to cancer [23,24]. In the present study, we evaluated the prognostic value of CBC-derived inflammatory scores and CTC EMT in 284 primary BC patients.

## 2. Results

The clinical characteristics of 284 chemo-naive invasive primary BC patients included in this study are shown in Table 1. The median age of the patients was 59 years, minimum 25 and maximum 84 years. Most of them were T1 (70.4%), low or intermediate grade (69.8%), with lymph node (LN) positivity of 35.7%. The HR status was mostly positive (83.8%), while the HER2 status was negative in the majority of cases (84.5%). The most frequent tumor subtype was luminal A present in 53.0% of patients, while 11% of patients were triple-negative BC.

Among 284 patients, 49 (17.3%) were CTC EMT-positive, which means that at least one of the EMT-inducing transcription factor (TF) gene transcripts was overexpressed in their CD45-depleted cell fraction. Blood sampling for CTCs assessment and CBC was performed before the treatment, usually on the day of surgery.

After assessing several different cut-offs including mean and median values as well as those published previously for primary BC [25,26,27,28], for further analysis we used the CBC-derived inflammation-based score cut-off values published previously for metastatic BC [20]. These were most strongly associated with the prognosis in CTC EMT-positive patients. Therefore, values of NLR ≥ 3, MLR ≥ 0.34, PLR ≥ 210, and SII ≥ 836 were considered as elevated. The frequency of patients with elevated NLR was 16.0%, for elevated MLR 15.7%, for elevated PLR 15.8%, and for elevated SII 15.5%. Neutrophils, monocytes, and platelets significantly positively correlated with each other: neutrophils and monocytes, *r* = 0.416; platelets and neutrophils, *r* = 0.222, platelets and monocytes, r = 0.236; in all cases, *p* < 0.001, although the last two correlations were rather weak.

### 2.1. Prognostic Role of Circulating Tumor Cells

**Figure 1:**
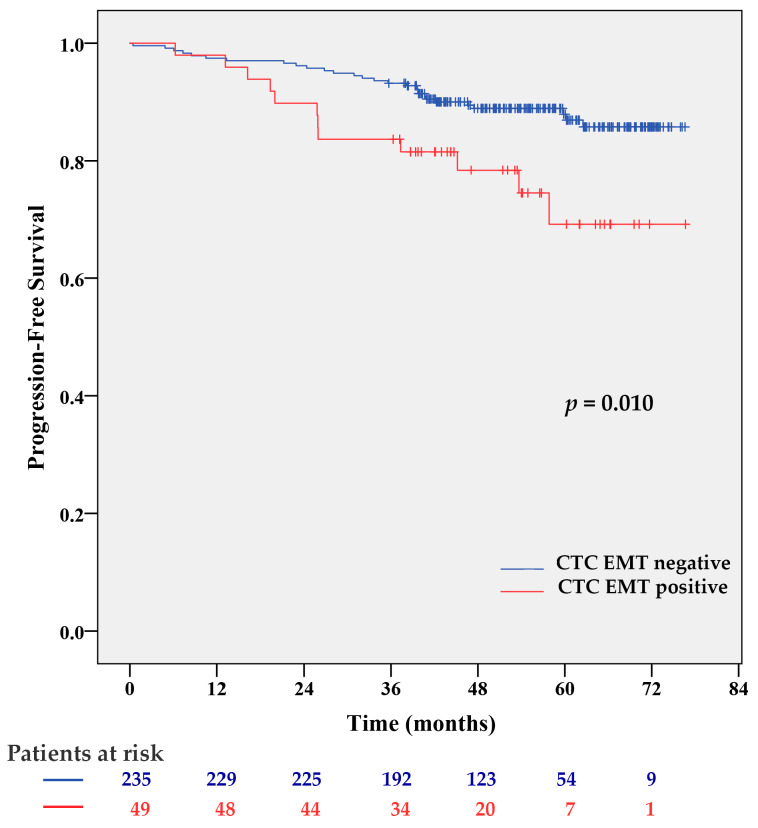
Kaplan–Meier PFS estimates for CTC EMT. CTC EMT-positive patients had significantly shorter progression-free survival (PFS) than CTC EMT-negative patients (*p* = 0.010 by Log-rank test).

At a mean follow-up time of 52.6 ± 15.1 months (range 0.4 to 76.7 months), 40 patients (14.1%) experienced progression-free survival (PFS), and 17 patients (6.3%) had died. Because of the immaturity of the OS data, only the results of the PFS analyses are presented. CTC EMT-positive patients had a significantly shorter PFS than CTC EMT-negative patients (*p* = 0.010 by Log-rank test) (Figure 1). CTC EMT positivity was associated with 2.4 times increased risk of disease progression (95% CI 1.20–4.66, *p* = 0.013). Two-year PFS was observed in 96% of CTC EMT-negative and 89% of CTC EMT-positive patients. This difference was more pronounced for five-year survival, which indicated that 90% of CTC EMT-negative patients remained without progression, in contrast to only 69% of CTC EMT-positive cases (Figure 1).

We assessed differences between clinical characteristics of CTC EMT-negative and CTC EMT-positive patients. No significant differences in clinical characteristics were identified between these two groups (Table 1).

### 2.2. Complete Blood Count-Derived Inflammation-Based Scores

**Figure 2:**
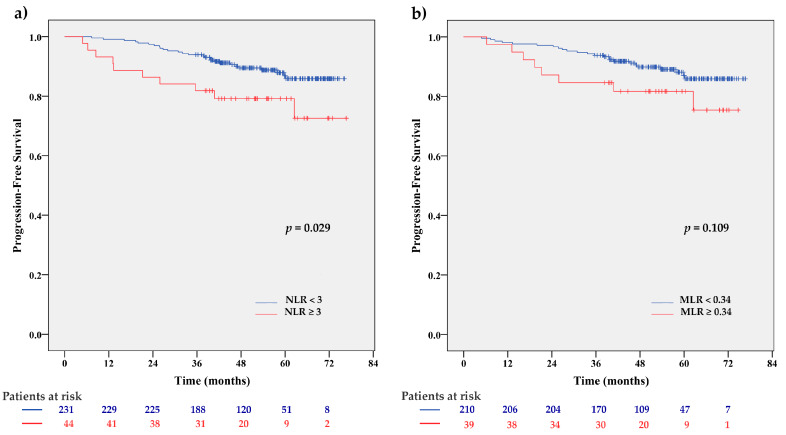
Kaplan–Meier PFS estimates for NLR (**a**) and MLR (**b**) status. PFS was significantly reduced in patients with NLR ≥ 3 compared to those with NLR < 3 (*p* = 0.029 by Log-rank test), while the difference was not significant for MLR (*p* = 0.109 by Log-rank test).

Except for NLR (HR = 2.20; 95% CI 1.07–4.55; *p* = 0.033), CBC-derived inflammation-based scores at the indicated cut-off values were not significantly associated with PFS. Patients with NLR ≥ 3 had shorter PFS compared to those with NLR < 3 (*p* = 0.029 by Log-rank test) (Figure 2).

In multivariate analysis, NLR ≥ 3, age over 50 years, high grade, lymph node positivity, and high (>20%) Ki-67 proliferation index were independently associated with PFS (Table 2).

Although at a cut-off 0.34 MLR did not demonstrate prognostic significance, the MLR alone was associated with PFS at a cut-off of 0.28 (mean value) (HR = 2.17; 95% CI 1.08–4.35; *p* = 0.029), therefore both NLR and MLR were included in subsequent analyses.

### 2.3. Increased Prognostic Value of CTC EMT in Combination with Complete Blood Count-Derived Inflammation-Based Scores

**Figure 3:**
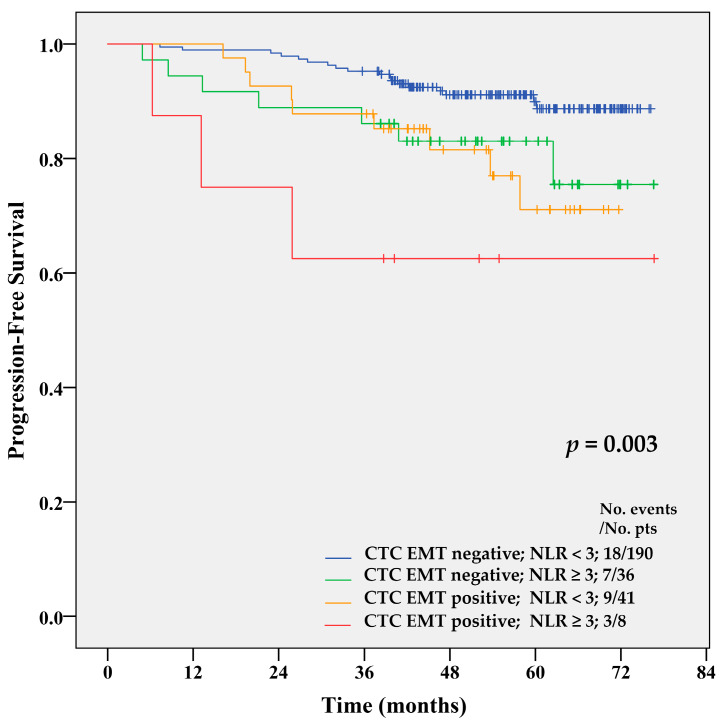
Kaplan–Meier PFS estimates for combinations of CTC EMT and NLR index. CTC EMT-positive patients with NLR ≥ 3 had shorter PFS compared to patients with any other combination of CTC EMT and NLR (*p* = 0.003 by Log-rank test).

In the overall population (*n* = 284), CTCs did not correlate with the percentage of monocytes, neutrophils, lymphocytes, or thrombocytes. However, the combination of CTC EMT with the NLR and MLR indexes significantly increased the prognostic value of CTC EMT for primary BC (*p* = 0.006 for NLR (cut-off 3) and *p* < 0.001 for MLR (cut-off 0.34) by univariate analysis). Patients with the presence of CTC EMT and elevated NLR had 6.2 times increased risk of progression (95% CI 1.83–21.18; *p* = 0.003) in comparison to those without CTC EMT and with low NLR. In patients with CTC EMT positivity but without elevated NLR, the risk was increased 2.6 times (1.18–5.85; *p* = 0.018), while the risk of those with increased NLR but without CTC EMT was not significantly raised (HR = 2.25, 95% CI 0.94–5.38). CTC EMT-positive patients with elevated NLR had shorter PFS compared to the patients with all other combinations (*p* = 0.003 by Log-rank test) (Figure 3). Two-year PFS was 95% for CTC EMT-negative, NLR-low patients, 86% for CTC EMT-negative, NLR-high patients, 88% for CTC EMT-positive, NLR-low patients, and 63% for CTC EMT-positive, NLR-high patients. Five-year PFS was 88%, 73%, 72%, and 63%, respectively, for these groups of BC patients.

Multivariate analysis using the Cox proportional hazard model confirmed a combination of CTC EMT positivity and NLR ≥ 3 as an independent risk factor for disease recurrence (Table 3). Among clinical variables, older age, high grade, lymph nodes positivity, and high proliferation index significantly increased the risk of disease recurrence.

**Figure 4:**
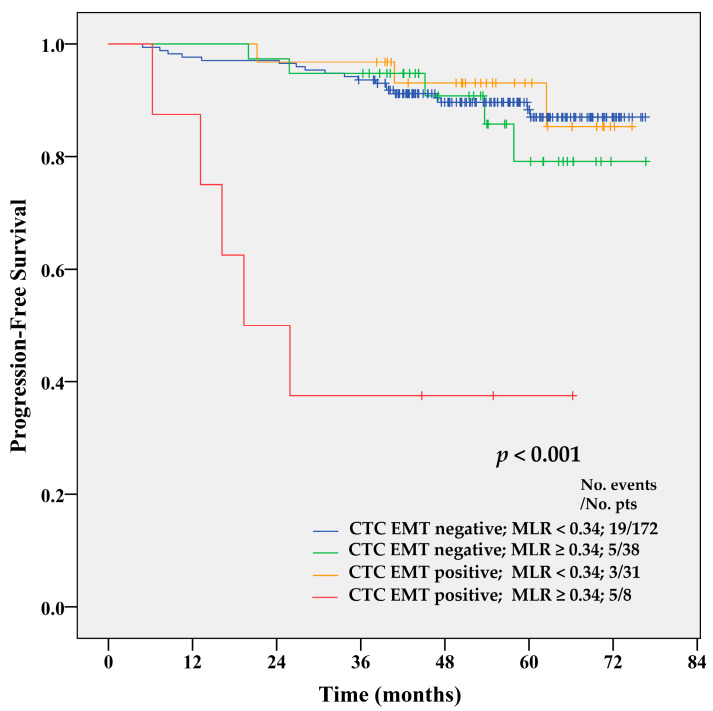
Kaplan–Meier PFS estimates for combinations of CTC EMT and MLR index. CTC EMT-positive patients with MLR ≥ 0.34 had shorter PFS compared to patients with all other combinations of CTC EMT and MLR (*p* < 0.001 by Log-rank test).

Although the MLR index was not significantly associated with PFS in univariate analysis using a cut-off of 0.34, its combination with the CTC EMT status significantly increased the prognostic value of CTC EMT. In univariate analysis, an MLR ≥ 0.34 in patients with CTC EMT was associated with significantly increased risk of disease recurrence (HR = 10.78, 95% CI 3.99–29.13; *p* < 0.001) and shorter PFS compared to all other combinations (*p* < 0.001 by Log-rank test) (Figure 4). Two-year PFS was 94%, 95%, 97%, and 38% for CTC EMT-negative, MLR-low patients, CTC EMT-negative, MLR-high patients, CTC EMT-positive, MLR-low patients, and CTC EMT-positive, MLR-high patients. Five-year PFS was 86%, 81%, 82% and 38% for the same groups of BC patients.

Multivariate Cox proportional hazard regression analysis (Table 4) was employed to analyze the effect of several covariates, including clinical predictor variables (listed in Table 1), on PFS. It confirmed the prognostic value of the CTC EMT positivity combined with the MLR as an independent risk factor for disease recurrence. Disease recurrence was 13.14 times more likely in patients with both CTC EMT positivity and elevated MLR (95% CI 4.35–39.67; *p* < 0.001) in comparison to CTC EMT-negative patients with low MLR. Among the tested clinical characteristics, older age, presence of positive lymph nodes, and high grade significantly increased the risk of disease recurrence. Surprisingly, higher T-stage lowered the risk, although with a borderline significance (*p* = 0.043).

Importantly, the results of the multivariate analysis did not confirm the significant result of the univariate analysis for an MLR cut-off of 0.28 in combination with the CTC EMT positivity.

## 3. Discussion

Currently, research is focused on the discovery of new cancer biomarkers to better determine patients’ clinical outcome, identify patients at risk, and recognize responders to treatment. With the introduction of novel techniques, liquid biopsy holds the potential for fulfilling these goals. Nevertheless, due to serious challenges regarding the specificity and sensitivity of the available assays, it is not yet used in routine clinical practice. The term “liquid biopsy” was originally introduced to define CTCs in peripheral blood [29]. Although CTCs and CTC clusters were proven prognostically valuable in several cancer types [30], technical and statistical concerns were initially raised about their validity in early-stage BC, where they are relatively rare [31]. Recently, an international expert panel proposed the clinical use of CTCs enumeration (cut-off ≥5 CTCs per 7.5 mL of blood using the FDA-cleared CellSearch System) as an important tool for the stratification of BC patients with stage IV disease [32]. Moreover, the pooled analysis of the prognostic significance of EpCAM^+^ CTCs from patients in stage I to III (CTCs threshold for non-metastatic patients >1 per 7.5 mL blood) showed the presence of CTCs as an independent prognostic factor for inferior disease-free survival (DFS), distant DFS, breast cancer-specific survival, and OS [33]. Even though these robust methods, based on the expression of epithelial markers, consistently confirmed the prognostic value of epithelial CTCs, emerging evidence suggests that CTCs are highly heterogeneous. In addition to epithelial CTCs, they consist also of CTC EMT, hybrid (epithelial/EMT+) tumor cells, irreversible EMT^+^ tumor cells, and circulating tumor stem cells [34]. Thus, the EpCAM-based approach can underestimate the significance of these non-epithelial tumor subtypes. CTC dissemination is strongly influenced by EMT, facilitating the invasion and intravasation of epithelial cancer cells into the bloodstream [35]. The mesenchymal CTC phenotype has been associated with unfavorable outcomes in primary and metastatic BC [19,36,37,38]. In our previous study, the prognostic value of CTC EMT was demonstrated in all clinical BC subtypes. However, it was most pronounced in the hormone receptor-positive and HER2-negative subgroups [19]. Papadaki et al. [36] surveyed 130 patients before and after first-line chemotherapy and showed that patients whose CTCs exhibited cancer stem cell (CSC) markers and partial EMT had decreased PFS. In HER2-negative patients, the presence of CSC^+^/partial-EMT^+^ CTCs was additionally associated with reduced OS. The authors hypothesized that CTCs that already underwent EMT represent a chemoresistant population and independently predict an unfavorable outcome. In other paper by Bulfoni et al. [39], the authors pointed out the importance of the fraction of CD45^−^ cells which co-express epithelial and mesenchymal markers, since this is the population of CTCs which is significantly associated with poorer PFS and OS. Current data suggest that CTC EMT assessment could become a valuable tool to refine patient prognosis determined by the use of EpCAM-based technologies [34,40]. Recently, Fischer et al., by using a Cre/CreER lineage-tracing reporter system to mark carcinoma cells that had undergone EMT activation, showed that mammary tumor cells could metastasize without activating EMT programs [41]. Results stirred up a debate [42] regarding the tools they used and pointed out the necessity to define correct criteria for claiming effective EMT cell tracing (cautious marker and gene/protein selection). Alternative models of dissemination, such as collective or cluster-based migration and invasion, were also proposed, which are beyond the traditional EMT/mesenchymal-to-epithelial (MET) view, so their role in the metastatic process should be further investigated as well.

A dynamic shift towards mesenchymal CTC populations correlated with BC treatment failure and disease progression, as reported by Yu and colleagues [43]. The authors showed that the increase in mesenchymal CTC populations during relapse is associated with the presence of tumor microemboli or multicellular CTC clusters in the blood. These clusters represent conglomerates of the tumor and other types of cells, including platelets, immune cells, and cancer-associated fibroblasts [30]. Heeke and colleagues demonstrated that CTCs interact closely with these cells to overcome biological and physical challenges posed by the circulation [44]. On the other side, pro-inflammatory cytokines and other immunomodulatory molecules can be produced by cancer cells to favor tumor growth, infiltration, and metastasis. Several soluble peripheral blood-based parameters are currently investigated as factors that reflect the host’s immune response. It has been suggested that a shift in the neutrophil, lymphocyte, or platelet count may be associated with a systemic inflammatory response in several types of solid tumors [45,46,47,48,49,50]. Although the CBC-derived inflammation-based scores cut-off values were not unified, published data confirm the association of elevated PLR and NLR with poor PFS and OS in BC [25,51,52]. A more recent meta-analysis addressed the prognostic value of NLR and PLR in a total of 39 studies comprising 17,079 patients, showing their association with poor OS and high risk of disease recurrence [53]. Herein, we assessed the prognostic value of four CBC-derived inflammation-based scores, namely, NLR, MLR, PLR, and SII, in 284 primary BC patients. Only NLR was significantly associated with PFS. Published PLR cut-off values ranged from 107 [54] to 292 [55]. However, none of the cut-offs tested was associated with PFS in our study. A systematic review and meta-analysis of Ethier et al. showed NLR cut-off values ranging from 1.9 to 5.0 [26]. The median cut-off value, the one most frequently associated with adverse outcomes, similarly to our data, was cut-off 3.0 among the reported 13 studies, followed by cut-off 2.5 in 10 studies. Similar results, showing that early-stage BC patients with high NLR were more prone to suffer a postoperative recurrence, were published by Geng and colleagues using a cut-off of 1.88 [56]. On the other hand, the prognostic impact of NLR was not confirmed in metastatic BC by Rubio and colleagues, who used the median value cut-off of 2.32 [57]. The ratio between lymphocytes and monocytes was in the majority of the studies reported as LMR and ranged in advanced epithelial cancers between 2.35 and 5.46. A high pretreatment LMR (cut-off value 3.0) was associated with favorable OS and PFS [27]. The MLR cut-off of 0.34 used in our study is equivalent to an LMR value of 3.0. However, in contrast to the MLR cut-off of 0.28 (equivalent to LMR 3.6), this value was not associated with PFS for MLR alone. An LMR > 4.8 was significantly associated with superior DFS in the study of Jia et al., although this association was not confirmed by multivariate analysis [28]. The prognostic potential of MLR was identified less frequently in primary BC. In a large cohort study, the elevated preoperative circulating absolute monocyte count was associated with shorter OS [58]. These ambiguous results, largely influenced by the diverse cut-off values, hinder the clinical application of CBC-derived inflammation-based scores.

Despite the low prognostic value of CBC-derived inflammation-based scores, with the only exception of NLR, we observed a strong association between adverse outcome and elevated NLR and MLR in CTC EMT-positive patients. Similar results were published recently for metastatic BC with ≥5 CTC per 7.5 mL of blood, showing that CTC positivity and elevated MLR are predictors of poor OS, although the prognostic roles of NLR, PLR, and SII were not confirmed by multivariate analysis [20]. The interaction between neutrophils and tumor cells is hypothesized to be mediated by VCAM-1 and results in the formation of CTC–neutrophil clusters endowed with a higher metastasis-forming potential compared to CTCs that do not interact with neutrophils [4]. Molecular analysis of CTCs in clusters displayed a marked enrichment in positive regulators of the cell cycle and DNA replication programs. The most frequently expressed genes in cluster-associated neutrophils encoded four cytokines, i.e., TNF-α, Oncostatin M-OSM, IL-1β, and IL-6. CTCs from CTC–neutrophil clusters most frequently expressed Colony Stimulating Factor 1 (CSF1), CSF3, TGF-β3, and IL-15, cytokines possibly involved in neutrophil stimulation [59,60]. In the mouse BC model, neutrophils were recognized as the main component and driver of metastatic establishment within the (pre-)metastatic lung microenvironment. Via neutrophil-derived leukotrienes, they aid the colonization of distant tissues through selective expansion of the sub-pool of cancer cells that retain high tumorigenic potential [58]. In small cell lung cancer, the interaction of CTCs with peripheral blood mononuclear cells in vitro resulted in monocyte–macrophage differentiation and was accompanied by enhanced malignant phenotype of tumor cells and attraction of angiogenic neutrophils [61]. Also in BC, tumor-associated macrophages derived from circulating monocytes were shown to play a critical role in disease progression by promoting angiogenesis, tumor cell migration, and metastasis, as well as immune evasion [62,63]. In the peripheral blood of metastatic BC patients, also circulating cancer-associated macrophage-like cells (CAMLs) were detected [64]. Their enumeration at baseline independently predicted disease progression and OS, especially in patients with ≥5 CTCs/7.5 mL blood.

Although CTC definition in cancer research represents a landmark, the increasing number of reports show various new subpopulations of CTCs, whose study will help to better characterize their prognostic role and clinical relevance in cancer. Dual-positive circulating cells, a curious phenomenon of CTC and macrophage fusion, were described recently (circulating cells expressing both epithelial and leukocyte markers) and were associated with increased growth and motility, enhanced invasion, and higher efficiency in metastasis formation (reviewed in [65,66]). This and other findings support the hypothesis that immune cells in the bloodstream can expand the metastatic potential of CTCs.

A cost-effective analysis and the accessibility of NLR and MLR qualify them as excellent laboratory biomarkers with significant prognostic value for PFS and OS when combined with CTC EMT information. However, the relatively small sample size, reflected in the small absolute number of events, is the major limitation of this study. Therefore, further large-scale prospective trials containing OS data are needed to verify the prognostic value of CBC-derived inflammation-based scores in CTC-negative and -positive BC patients.

## 4. Materials and Methods

### 4.1. Study Population

This case–control study included 284 chemo-naive, stage I–III invasive primary BC patients treated by surgery between March 2012 and February 2015 (Protocol TRU-SK 002; Chair: M. Mego). The selection of the participants was based on the availability of CTC data. The median age of the included patients was 59.1 (range 24.7–83.5) years. Peripheral blood was collected from each participant. The exclusion criterion was concurrent malignancy other than non-melanoma skin cancer in the previous 5 years. In total, 209 (73.6%) of the patients were older than 50 years, 84 patients (29.6%) were diagnosed with stage T2 or T3 BC, 100 patients (35.7%) were lymph node-positive (N+), and 50 (17.6%) were presented tumor cells inside the capillary lumens of either the lymphatic or microvascular drainage system (lymphovascular invasion, LVI). The study group consisted of 242 (85.2%) invasive ductal carcinomas (IDCs) and 42 (14.8%) other clinical subtypes including invasive lobular, tubular, or mucinous carcinomas. Histological grade 3 (high grade) was diagnosed in 84 (29.6%) of the patients, hormone receptor (HR) negativity in 46 (16.2%), HER2 positivity in 44 (15.5%), and Ki-67 proliferation >20% in 109 (38.5%). In addition, 105 patients (37.1%) were p53-positive, and 82 (28.9%) were bcl2-negative. The histological subtypes consisted of 150 (53%) luminal A, 58 (20.5%) luminal B, 44 (15.5%) HER2-positive, and 31 (11%) triple-negative. The Institutional Review Board of the National Cancer Institute of Slovakia approved this study; the approval number is: TRUSK002. Peripheral blood samples of 60 age-matched healthy women, who were recruited and consented to the Institutional Review board-approved protocol, were also analyzed. Written informed consent was obtained from all participants before study enrolment.

### 4.2. Identification of Gene Transcripts in CD45-Enriched Subsets

For CTCs enrichment, 9 mL of peripheral blood was subjected to CD45 depletion using RossetteSep^TM^ Human CD45 Depletion Cocktail (StemCell Technologies, Vancouver, BC, Canada), as published previously [67,68]. CD45-depleted cells were mixed with TRIzolVR LS Reagent (Invitrogen Corporation, Carlsbad, CA, USA). The isolated RNA was treated by DNAse (Ambion, Austin, TX, USA) and stored at 80 °C. The RNA concentrations were determined by absorbance readings at 260 nm. The RNA extracted from HELA, HCT116, MCF-7, and MDA-MB-231 cell lines was used as positive control. Reverse transcription was carried out with the cDNA archive kit (Applied Biosystems, Foster City, CA, USA).

Quantitative RT-PCR was used to detect EMT-associated TF (*TWIST1*, *SNAI1*, *SLUG*, and *ZEB1*) gene transcripts. In brief, 2.5 μL of cDNA was used in 25 μL reaction containing 12.5 μL of QuantiFast Probe RT-PCR Kit Master Mix (Qiagen GmbH, Hilden, Germany), 0.25 μL QuantiFast RT Mix (Qiagen GmbH, Hilden, Germany), 8.5 μL water, and 1.25 μL primers. TaqMan assays were obtained from LifeTechnologies (Carlsbad, CA, USA): *TWIST1*: Hs00361186_m1; *SNAI1*: Hs00195591_m1; *SLUG*: Hs00161904_m1; *ZEB1*: Hs01566408_m1; *GAPDH*: Hs99999905_m1. All samples were analyzed in triplicates. Amplification was performed on an Eppendorf RealPlex RT-PCR system (Eppendorf AG, Hamburg, Germany), using the following cycling program: 95 °C for 10 min; 40 cycles at 95 °C for 15 s and 60 °C for 60 s. Calibrator samples were run with every plate to ensure consistency of the PCR. Fluorescence was detected between 0 and 40 cycles for the control and marker genes in single-plex reactions, allowing for the deduction of the cycles at threshold (Ct) value for each product. The expression of the genes of interest was calibrated against the expression of the housekeeping gene GAPDH. Target cDNA was quantified using a semiquantitative delta-Ct approach with the formula: 2 ^ (Ct target − Ct GAPDH).

The Ct values of 60 healthy donors were used as a cut-off to determine CTC positivity. The highest expression levels of EMT-associated TF gene transcripts relative to that of GAPDH in the controls were 2.0 × 10^−4^, 1 × 10^−2^, and 2.2 × 10^−2^ for *TWIST1*, *SNAI1*, and *ZEB1*, respectively. The *SLUG* transcripts were not detected in any of the samples from healthy donors. These highest expression levels of the EMT-associated TF gene transcripts relative to that of GAPDH in the controls were set as cut-off values; patient samples with higher expression of EMT-associated TF gene transcripts were considered as CTC EMT-positive.

### 4.3. Complete Blood Count and CBC-Derived Inflammation-Based Scores

CBC and leukocyte differential data were collected from patient health records. NLR was calculated as neutrophils/lymphocytes count, MLR as monocytes/lymphocytes count, and PLR as platelets/lymphocytes count at the baseline data. SII, based on platelet, neutrophil, and lymphocyte counts, was calculated using the formula platelets × neutrophils/lymphocytes. For CBC-derived inflammation-based scores, cut-off values identical with those published previously for metastatic BC patients were used [20].

### 4.4. Statistical Analysis

Descriptive statistics were used to summarize patient clinical characteristics (frequency for categorical variables, median and range for continuous variables). The Chi-Square test or Fisher’s exact test were used to assess the association between categorical variables. For continuous variables, the normality of distribution was tested by the Kolmogorov–Smirnov and Shapiro–Wilk tests. Normally distributed data were tested by Student’s *t*-test or analysis of variance (ANOVA) with Bonferroni’s corrections. Non-normally distributed data were tested by non-parametric Mann–Whitney U or Kruskal–Wallis H tests. Pearson’s or Spearman’s correlation coefficients were calculated depending on the normality of distribution.

PFS was defined as the time interval from the date of sampling (usually date of surgery) to the date of disease recurrence, death, or last follow-up. PFS was estimated by the Kaplan–Meier product-limit method and compared between groups with the Log-rank test. A multivariate Cox proportional hazard model using a stepwise regression procedure was applied to evaluate the prognostic significance of CTC and/or individual CBC-derived inflammation-based scores after adjustment for clinicopathological variables as defined in Table 1 (age, Ki-67, HR and HER2 status, tumor size, grade, Tp53 and bcl2 status). A backward model selection was conducted, and the final fitted model is presented.

The univariate Cox regression model was used to investigate prognostic indicators of PFS and to estimate the hazard ratios and their 95% confidence intervals. Statistical differences were considered significant when *p* < 0.05. All analyses were performed using IBM SPSS statistics version 23.0 software for Windows (IBM).

## 5. Conclusions

The role of immune cells during hematogenous dissemination in BC remains largely uncharacterized. Here, we identified for the first time the link between the presence of mesenchymal CTCs and CBC-derived inflammation-based scores (NLR, MLR) in primary BC. Our results confirm the hypothesis that immune cells in the bloodstream can expand the metastatic potential of CTCs. A deeper understanding of this interaction may provide an opportunity for the development of new therapeutic strategies.

## Figures and Tables

**Table 1:** Clinical characteristics of all CTC EMT-negative and -positive patients.

Variables	Categories	All Patients (*n* = 284)	CTC EMT Negative (*n* = 235)	CTC EMT Positive (*n* = 49)	*p* ^#^
Age (years) Median (range)		59.1 (24.66–83.49)	58.8 (24.7–83.36)	59.5 (33.5–83.49)	0.323
Age (years)	≤50	75 (26.4)	67 (28.5)	8 (16.3)	0.078
	>50	209 (73.6)	168 (71.5)	41 (83.7)	
T-stage	T1	200 (70.4)	168 (71.5)	32 (65.3)	0.388
	T2 and more	84 (29.6)	67 (28.5)	17 (34.7)	
Histology	IDC	242 (85.2)	199 (84.7)	43 (87.8)	0.581
	Others	42 (14.8)	36 (15.3)	6 (12.2)	
Grade	Low and intermediate	194 (69.8)	164 (70.4)	30 (66.7)	0.619
	High	84 (29.6)	69 (29.6)	15 (33.3)	
N-stage	N0	180 (64.3)	151 (64.8)	29 (61.7)	0.685
	N+	100 (35.7)	82 (35.2)	18 (38.3)	
LVI	Absent	234 (82.4)	196 (83.4)	38 (77.6)	0.328
	Present	50 (17.6)	39 (16.6)	11 (22.4)	
HR status ^$^	Negative	46 (16.2)	37 (15.7)	9 (18.4)	0.650
	Positive	238 (83.8)	198 (84.3)	40 (81.6)	
HER2 status	Negative	240 (84.5)	200 (85.1)	40 (81.6)	0.541
	Amplified	44 (15.5)	35 (14.9)	9 (18.4)	
p53	Negative	178 (62.9)	144 (61.5)	34 (69.4)	0.301
	Positive	105 (37.1)	90 (38.5)	15 (30.6)	
bcl2	Negative	82 (28.9)	64 (27.2)	18 (36.7)	0.182
	Positive	202 (71.1)	171 (72.8)	31 (63.3)	
Ki-67	<20%	174 (61.5)	148 (63.2)	26 (53.1)	0.183
	>20%	109 (38.5)	86 (36.8)	23 (46.9)	
Tumor subtypes	Luminal A	150 (53.0)	127 (54.3)	23 (46.9)	0.752
	Luminal B	58 (20.5)	46 (19.7)	12 (24.5)	
	HER2-positive	44 (15.5)	35 (15.0)	9 (18.4)	
	Triple-negative	31 (11.0)	26 (11.1)	5 (10.2)	
NLR	<3	231 (84.0)	190 (84.1)	41 (83.7)	0.945
	≥3	44 (16.0)	36 (15.9)	8 (16.3)	
MLR	<0.34	(210) 84.3	172 (84.7)	38 (82.6)	0.721
	≥0.34	(39) 15.7	31 (15.3)	8 (17.4)	
PLR	<210	223 (84.2)	183 (84.3)	40(83.3)	0.846
	≥210	42 (15.8)	34 (15.7)	8 (16.7)	
SII	<836	221 (77.8)	180 (82.9)	41 (85.4)	0.678
	≥836	44 (15.5)	37 (17.1)	7 (14.6)	

The total number of samples analyzed in the study was *n* = 284; only cases with valid information on individual variables were included in the table; ^#^ difference between CTC EMT-positive and -negative patients; ^$^ negative for both or positive for either. Abbreviations: IDC, invasive ductal carcinoma; LVI, lymphovascular invasion; HR, hormonal receptor, NLR, neutrophil-to-lymphocyte ratio; PLR, platelet-to-lymphocyte ratio; MLR, monocyte-to-lymphocyte ratio; SII, systemic immune-inflammation index.

**Table 2:** Cox proportional hazard regression analysis for the association between NLR, clinical predictor variables, and PFS.

Variables	HR	95% CI	*p*
NLR ≥ 3	2.45	1.17–5.12	0.017
Age > 50	3.15	1.11–8.96	0.031
High grade	2.36	1.12–4.96	0.024
N+	2.18	1.11–4.28	0.023
Ki-67 > 20%	3.03	1.38–6.65	0.006

**Table 3:** Cox proportional hazard regression analysis for the association between CTC EMT and NLR status, clinical predictor variables, and PFS.

Variable	HR	95% CI	*p*
CTC EMT-negative, NLR < 3			0.006
CTC EMT-negative, NLR ≥ 3	2.30	0.95–5.57	0.065
CTC EMT-positive, NLR < 3	2.06	0.87–4.85	0.099
CTC EMT-positive, NLR ≥ 3	8.60	2.35–31.48	0.001
Age > 50	2.90	1.01–8.36	0.049
High grade	2.58	1.20–5.53	0.015
N+	2.46	1.24–4.87	0.010
Ki67 > 20	2.79	1.27–6.11	0.010

**Table 4:** Cox proportional hazard regression analysis for the association between CTC EMT and MLR status, clinical predictor variables, and PFS.

Variable	HR	95% CI	*p*
CTC EMT-negative, MLR < 0.34			<0.001
CTC EMT-negative, MLR ≥ 0.34	1.00	0.33–2.98	0.997
CTC EMT-positive, MLR < 0.34	1.12	0.33–3.84	0.852
CTC EMT-positive, MLR ≥ 0.34	13.14	4.35–39.67	<0.001
Age > 50	3.35	1.00–11.22	0.050
T2 and more	0.41	0.17–0.97	0.043
High grade	3.93	1.85–8.34	<0.001
N+	3.36	1.56–7.22	0.002

## References

[1] Harbeck N., Penault-Llorca F., Cortes J., Gnant M., Houssami N., Poortmans P., Ruddy K., Tsang J., Cardoso F. (2019). Breast cancer. Nat. Rev. Dis. Prim..

[2] Arciero C.A., Styblo T.M. (2018). Clinically Established Prognostic Factors in Breast Cancer. The Breast.

[3] Greten F.R., Grivennikov S.I. (2019). Inflammation and Cancer: Triggers, Mechanisms, and Consequences. Immunity.

[4] Saini M., Szczerba B.M., Aceto N. (2019). Circulating Tumor Cell-Neutrophil Tango along the Metastatic Process. Cancer Res..

[5] Coffelt S.B., Wellenstein M.D., de Visser K.E. (2016). Neutrophils in cancer: Neutral no more. Nat. Rev. Cancer.

[6] Finisguerra V., Di Conza G., Di Matteo M., Serneels J., Costa S., Thompson A.A., Wauters E., Walmsley S., Prenen H., Granot Z. (2015). MET is required for the recruitment of anti-tumoural neutrophils. Nature.

[7] Galdiero M.R., Bonavita E., Barajon I., Garlanda C., Mantovani A., Jaillon S. (2013). Tumor associated macrophages and neutrophils in cancer. Immunobiology.

[8] Sagiv J.Y., Michaeli J., Assi S., Mishalian I., Kisos H., Levy L., Damti P., Lumbroso D., Polyansky L., Sionov R.V. (2015). Phenotypic diversity and plasticity in circulating neutrophil subpopulations in cancer. Cell Rep..

[9] Ponzetta A., Carriero R., Carnevale S., Barbagallo M., Molgora M., Perucchini C., Magrini E., Gianni F., Kunderfranco P., Polentarutti N. (2019). Neutrophils Driving Unconventional T Cells Mediate Resistance against Murine Sarcomas and Selected Human Tumors. Cell.

[10] Fridlender Z.G., Albelda S.M. (2012). Tumor-associated neutrophils: Friend or foe?. Carcinogenesis.

[11] Gregory A.D., Houghton A.M. (2011). Tumor-associated neutrophils: New targets for cancer therapy. Cancer Res..

[12] Piccard H., Muschel R.J., Opdenakker G. (2012). On the dual roles and polarized phenotypes of neutrophils in tumor development and progression. Crit. Rev. Oncol. Hematol..

[13] Kowanetz M., Wu X., Lee J., Tan M., Hagenbeek T., Qu X., Yu L., Ross J., Korsisaari N., Cao T. (2010). Granulocyte-colony stimulating factor promotes lung metastasis through mobilization of Ly6G+Ly6C+ granulocytes. Proc. Natl. Acad. Sci. USA.

[14] Granot Z., Henke E., Comen E.A., King T.A., Norton L., Benezra R. (2011). Tumor entrained neutrophils inhibit seeding in the premetastatic lung. Cancer Cell.

[15] Huh S.J., Liang S., Sharma A., Dong C., Robertson G.P. (2010). Transiently entrapped circulating tumor cells interact with neutrophils to facilitate lung metastasis development. Cancer Res..

[16] Pantel K., Speicher M.R. (2016). The biology of circulating tumor cells. Oncogene.

[17] Mego M., Cierna Z., Janega P., Karaba M., Minarik G., Benca J., Sedlackova T., Sieberova G., Gronesova P., Manasova D. (2015). Relationship between circulating tumor cells and epithelial to mesenchymal transition in early breast cancer. BMC Cancer.

[18] Mego M., Gao H., Lee B.N., Cohen E.N., Tin S., Giordano A., Wu Q., Liu P., Nieto Y., Champlin R.E. (2012). Prognostic Value of EMT-Circulating Tumor Cells in Metastatic Breast Cancer Patients Undergoing High-Dose Chemotherapy with Autologous Hematopoietic Stem Cell Transplantation. J. Cancer.

[19] Mego M., Karaba M., Minarik G., Benca J., Silvia J., Sedlackova T., Manasova D., Kalavska K., Pindak D., Cristofanilli M. (2019). Circulating Tumor Cells with Epithelial-to-mesenchymal Transition Phenotypes Associated With Inferior Outcomes in Primary Breast Cancer. Anticancer. Res..

[20] De Giorgi U., Mego M., Scarpi E., Giordano A., Giuliano M., Valero V., Alvarez R.H., Ueno N.T., Cristofanilli M., Reuben J.M. (2019). Association between circulating tumor cells and peripheral blood monocytes in metastatic breast cancer. Ther. Adv. Med. Oncol..

[21] Olingy C.E., Dinh H.Q., Hedrick C.C. (2019). Monocyte heterogeneity and functions in cancer. J. Leukoc. Biol..

[22] Wang L., Simons D.L., Lu X., Tu T.Y., Avalos C., Chang A.Y., Dirbas F.M., Yim J.H., Waisman J., Lee P.P. (2020). Breast cancer induces systemic immune changes on cytokine signaling in peripheral blood monocytes and lymphocytes. EBioMedicine.

[23] Zhong J.H., Huang D.H., Chen Z.Y. (2017). Prognostic role of systemic immune-inflammation index in solid tumors: A systematic review and meta-analysis. Oncotarget.

[24] Huszno J., Kolosza Z. (2019). Prognostic value of the neutrophil-lymphocyte, platelet-lymphocyte and monocyte-lymphocyte ratio in breast cancer patients. Oncol. Lett..

[25] Ethier J.-L., Desautels D., Templeton A., Shah P.S., Amir E. (2017). Prognostic role of neutrophil-to-lymphocyte ratio in breast cancer: A systematic review and meta-analysis. Breast Cancer Res..

[26] Cuello-Lopez J., Fidalgo-Zapata A., Lopez-Agudelo L., Vasquez-Trespalacios E. (2018). Platelet-to-lymphocyte ratio as a predictive factor of complete pathologic response to neoadjuvant chemotherapy in breast cancer. PLoS ONE.

[27] Mao Y., Chen D., Duan S., Zhao Y., Wu C., Zhu F., Chen C., Chen Y. (2018). Prognostic impact of pretreatment lymphocyte-to-monocyte ratio in advanced epithelial cancers: A meta-analysis. Cancer Cell Int..

[28] Jia W., Wu J., Jia H., Yang Y., Zhang X., Chen K., Su F. (2015). The Peripheral Blood Neutrophil-To-Lymphocyte Ratio Is Superior to the Lymphocyte-To-Monocyte Ratio for Predicting the Long-Term Survival of Triple-Negative Breast Cancer Patients. PLoS ONE.

[29] Pantel K., Alix-Panabières C. (2010). Circulating tumour cells in cancer patients: Challenges and perspectives. Trends Mol. Med..

[30] Fabisiewicz A., Grzybowska E. (2017). CTC clusters in cancer progression and metastasis. Med. Oncol..

[31] Thery L., Meddis A., Cabel L., Proudhon C., Latouche A., Pierga J.-Y., Bidard F.-C. (2019). Circulating tumor cells in early breast cancer. JNCI Cancer Spectr..

[32] Cristofanilli M., Pierga J.Y., Reuben J., Rademaker A., Davis A.A., Peeters D.J., Fehm T., Nole F., Gisbert-Criado R., Mavroudis D. (2019). The clinical use of circulating tumor cells (CTCs) enumeration for staging of metastatic breast cancer (MBC): International expert consensus paper. Crit. Rev. Oncol. Hematol..

[33] Janni W.J., Rack B., Terstappen L.W., Pierga J.-Y., Taran F.-A., Fehm T., Hall C., De Groot M.R., Bidard F.-C., Friedl T.W. (2016). Pooled analysis of the prognostic relevance of circulating tumor cells in primary breast cancer. Clin. Cancer Res..

[34] Grover P.K., Cummins A.G., Price T.J., Roberts-Thomson I.C., Hardingham J.E. (2014). Circulating tumour cells: The evolving concept and the inadequacy of their enrichment by EpCAM-based methodology for basic and clinical cancer research. Ann. Oncol..

[35] Jie X.-X., Zhang X.-Y., Xu C.-J. (2017). Epithelial-to-mesenchymal transition, circulating tumor cells and cancer metastasis: Mechanisms and clinical applications. Oncotarget.

[36] Papadaki M.A., Stoupis G., Theodoropoulos P.A., Mavroudis D., Georgoulias V., Agelaki S. (2019). Circulating Tumor Cells with Stemness and Epithelial-to-Mesenchymal Transition Features Are Chemoresistant and Predictive of Poor Outcome in Metastatic Breast Cancer. Mol. Cancer Ther..

[37] Zhang S., Wu T., Peng X., Liu J., Liu F., Wu S., Liu S., Dong Y., Xie S., Ma S. (2017). Mesenchymal phenotype of circulating tumor cells is associated with distant metastasis in breast cancer patients. Cancer Manag. Res..

[38] Guan X., Ma F., Li C., Wu S., Hu S., Huang J., Sun X., Wang J., Luo Y., Cai R. (2019). The prognostic and therapeutic implications of circulating tumor cell phenotype detection based on epithelial–mesenchymal transition markers in the first-line chemotherapy of HER2-negative metastatic breast cancer. Cancer Commun..

[39] Bulfoni M., Gerratana L., Del Ben F., Marzinotto S., Sorrentino M., Turetta M., Scoles G., Toffoletto B., Isola M., Beltrami C.A. (2016). In patients with metastatic breast cancer the identification of circulating tumor cells in epithelial-to-mesenchymal transition is associated with a poor prognosis. Breast Cancer Res..

[40] Hayes D.F., Cristofanilli M., Budd G.T., Ellis M.J., Stopeck A., Miller M.C., Matera J., Allard W.J., Doyle G.V., Terstappen L.W. (2006). Circulating tumor cells at each follow-up time point during therapy of metastatic breast cancer patients predict progression-free and overall survival. Clin. Cancer Res..

[41] Fischer K.R., Durrans A., Lee S., Sheng J., Li F., Wong S.T., Choi H., El Rayes T., Ryu S., Troeger J. (2015). Epithelial-to-mesenchymal transition is not required for lung metastasis but contributes to chemoresistance. Nature.

[42] Ye X., Brabletz T., Kang Y., Longmore G.D., Nieto M.A., Stanger B.Z., Yang J., Weinberg R.A. (2017). Upholding a role for EMT in breast cancer metastasis. Nature.

[43] Yu M., Bardia A., Wittner B.S., Stott S.L., Smas M.E., Ting D.T., Isakoff S.J., Ciciliano J.C., Wells M.N., Shah A.M. (2013). Circulating breast tumor cells exhibit dynamic changes in epithelial and mesenchymal composition. Science.

[44] Heeke S., Mograbi B., Alix-Panabières C., Hofman P. (2019). Never Travel Alone: The Crosstalk of Circulating Tumor Cells and the Blood Microenvironment. Cells.

[45] Coussens L.M., Werb Z. (2002). Inflammation and cancer. Nature.

[46] Walsh S.R., Cook E.J., Goulder F., Justin T.A., Keeling N.J. (2005). Neutrophil-lymphocyte ratio as a prognostic factor in colorectal cancer. J. Surg. Oncol..

[47] Cao J., Zhu X., Zhao X., Li X.F., Xu R. (2016). Neutrophil-to-Lymphocyte Ratio Predicts PSA Response and Prognosis in Prostate Cancer: A Systematic Review and Meta-Analysis. PLoS ONE.

[48] Sarraf K.M., Belcher E., Raevsky E., Nicholson A.G., Goldstraw P., Lim E. (2009). Neutrophil/lymphocyte ratio and its association with survival after complete resection in non–small cell lung cancer. J. Thorac. Cardiovasc. Surg..

[49] Wei B., Yao M., Xing C., Wang W., Yao J., Hong Y., Liu Y., Fu P. (2016). The neutrophil lymphocyte ratio is associated with breast cancer prognosis: An updated systematic review and meta-analysis. Onco Targets Ther..

[50] Suppan C., Bjelic-Radisic V., La Garde M., Groselj-Strele A., Eberhard K., Samonigg H., Loibner H., Dandachi N., Balic M. (2015). Neutrophil/Lymphocyte ratio has no predictive or prognostic value in breast cancer patients undergoing preoperative systemic therapy. BMC Cancer.

[51] Zhang M., Huang X.-Z., Song Y.-X., Gao P., Sun J.-X., Wang Z.-N. (2017). High platelet-to-lymphocyte ratio predicts poor prognosis and clinicopathological characteristics in patients with breast cancer: A meta-analysis. BioMed Res. Int..

[52] Hernandez C.M., Madrona A.P., Vazquez P.G., Fernández P.G., Merino G.R., Romero J.A., Paricio P.P. (2018). Usefulness of lymphocyte-to-monocyte, neutrophil-to-monocyte and neutrophil-to-lymphocyte ratios as prognostic markers in breast cancer patients treated with neoadjuvant chemotherapy. Clin. Transl. Oncol..

[53] Guo W., Lu X., Liu Q., Zhang T., Li P., Qiao W., Deng M. (2019). Prognostic value of neutrophil-to-lymphocyte ratio and platelet-to-lymphocyte ratio for breast cancer patients: An updated meta-analysis of 17079 individuals. Cancer Med..

[54] Yao M., Liu Y., Jin H., Liu X., Lv K., Wei H., Du C., Wang S., Wei B., Fu P. (2014). Prognostic value of preoperative inflammatory markers in Chinese patients with breast cancer. Onco Targets Ther..

[55] Krenn-Pilko S., Langsenlehner U., Thurner E.M., Stojakovic T., Pichler M., Gerger A., Kapp K.S., Langsenlehner T. (2014). The elevated preoperative platelet-to-lymphocyte ratio predicts poor prognosis in breast cancer patients. Br. J. Cancer.

[56] Geng S.K., Fu S.M., Fu Y.P., Zhang H.W. (2018). Neutrophil to lymphocyte ratio is a prognostic factor for disease free survival in patients with breast cancer underwent curative resection. Medicine (Baltimore).

[57] Ivars Rubio A., Yufera J.C., de la Morena P., Fernández Sánchez A., Navarro Manzano E., García Garre E., García Martinez E., Marín Zafra G., Sánchez Cánovas M., García Torralba E. (2019). Neutrophil-lymphocyte ratio in metastatic breast cancer is not an independent predictor of survival, but depends on other variables. Sci. Rep..

[58] Wen J., Ye F., Huang X., Li S., Yang L., Xiao X., Xie X. (2015). Prognostic Significance of Preoperative Circulating Monocyte Count in Patients with Breast Cancer: Based on a Large Cohort Study. Medicine (Baltimore).

[59] Szczerba B.M., Castro-Giner F., Vetter M., Krol I., Gkountela S., Landin J., Scheidmann M.C., Donato C., Scherrer R., Singer J. (2019). Neutrophils escort circulating tumour cells to enable cell cycle progression. Nature.

[60] Wculek S.K., Malanchi I. (2015). Neutrophils support lung colonization of metastasis-initiating breast cancer cells. Nature.

[61] Hamilton G., Rath B., Klameth L., Hochmair M.J. (2016). Small cell lung cancer: Recruitment of macrophages by circulating tumor cells. Oncoimmunology.

[62] Wyckoff J.B., Wang Y., Lin E.Y., Li J.F., Goswami S., Stanley E.R., Segall J.E., Pollard J.W., Condeelis J. (2007). Direct visualization of macrophage-assisted tumor cell intravasation in mammary tumors. Cancer Res..

[63] Williams C.B., Yeh E.S., Soloff A.C. (2016). Tumor-associated macrophages: Unwitting accomplices in breast cancer malignancy. NPJ Breast Cancer.

[64] Mu Z., Wang C., Ye Z., Rossi G., Sun C., Li L., Zhu Z., Yang H., Cristofanilli M. (2017). Prognostic values of cancer associated macrophage-like cells (CAML) enumeration in metastatic breast cancer. Breast Cancer Res. Treat..

[65] Reduzzi C., Vismara M., Gerratana L., Silvestri M., De Braud F., Raspagliesi F., Verzoni E., Di Cosimo S., Locati L.D., Cristofanilli M. (2020). The curious phenomenon of dual-positive circulating cells: Longtime overlooked tumor cells. Semin. Cancer Biol..

[66] Gast C.E., Silk A.D., Zarour L., Riegler L., Burkhart J.G., Gustafson K.T., Parappilly M.S., Roh-Johnson M., Goodman J.R., Olson B. (2018). Cell fusion potentiates tumor heterogeneity and reveals circulating hybrid cells that correlate with stage and survival. Sci. Adv..

[67] Cierna Z., Mego M., Janega P., Karaba M., Minarik G., Benca J., Sedlácková T., Cingelova S., Gronesova P., Manasova D. (2014). Matrix metalloproteinase 1 and circulating tumor cells in early breast cancer. BMC Cancer.

[68] Mego M., Mani S.A., Lee B.N., Li C., Evans K.W., Cohen E.N., Gao H., Jackson S.A., Giordano A., Hortobagyi G.N. (2012). Expression of epithelial-mesenchymal transition-inducing transcription factors in primary breast cancer: The effect of neoadjuvant therapy. Int. J. Cancer.

